# Flufenamic acid abrogates chronic swim stress‐induced depressive‐like symptoms via inhibiting neuroinflammation

**DOI:** 10.1002/alz70855_098780

**Published:** 2025-12-23

**Authors:** Garima Sharma, Monika Kadian, Anushka Vashishth, Anil Kumar

**Affiliations:** ^1^ University institute of pharmaceutical sciences, Panjab University, Chandigarh, Chandigarh, India

## Abstract

**Background:**

In recent years neuroinflammation hypothesis has emerged as a major player in major depressive disorder. Oxido‐inflammatory stress is known to activate inflammasome NLRP3, an adaptor protein that has been implicated in the pathogenesis of depression.

**Objective:**

The current investigation was created with the aim of investigating the effect of flufenamic acid in experimental model of depression like behavour.

**Method:**

In the current study male balb/c mice were used. animals were exposed to forced swim stress for 21 days. Flufenamic acid (10 and 20 mg/kg, per oral) alone and in combination with flouxetine was used as treatment. Further behavioural (forced swim test, and sucrose preference test), Biochemicals (oxidative stress and anti‐oxidant levels), mitochondrial enzyme activities, corticosterone levels and monoamine and their metabolite levels (Norepinephrine, Seretonin, Dopamine, Homovanillic acid, Dihydroxy phenyl aceioc acid and 5‐hydroxyindole acetic acid) were assessed

**Results:**

forced swim stress mice showed depression‐like behavioural alterations characterized by increased immobility time in forced swim test and decreased sucrose preference. Flufenamic acid significantly ameliorated depression‐like behavioural alterations. Flufenamic acid also significantly restored antioxidant enzymes, and increased oxidative stress in cerebral cortex and hippocampus showing its anti‐stress and antioxidant property, restored mitochondrial enzyme activities, regained corticosterone levels and attenuated altered monoamine levels and histopathological alterations in cortex and hippocampus.

**Conclusion:**

the current study demonstrates the neuroprotective potential of flufenamic acid in experimental model of depression like behavour.

